# Using dense seismo-acoustic network to provide timely warning of the 2019 paroxysmal Stromboli eruptions

**DOI:** 10.1038/s41598-021-93942-x

**Published:** 2021-07-14

**Authors:** A. Le Pichon, C. Pilger, L. Ceranna, E. Marchetti, G. Lacanna, V. Souty, J. Vergoz, C. Listowski, B. Hernandez, G. Mazet-Roux, A. Dupont, P. Hereil

**Affiliations:** 1 CEA/DAM/DIF, 91297 Arpajon, France; 2 BGR, B4.3, 30655 Hannover, Germany; 3 Department of Earth Sciences, University of Firenze, 50121 Firenze, Italy; 4 Meteo France, Toulouse VAAC, 31057 Toulouse, France

**Keywords:** Volcanology, Acoustics, Imaging techniques, Natural hazards, Volcanology, Geophysics

## Abstract

Stromboli Volcano is well known for its persistent explosive activity. On July 3rd and August 28th 2019, two paroxysmal explosions occurred, generating an eruptive column that quickly rose up to 5 km above sea level. Both events were detected by advanced local monitoring networks operated by Istituto Nazionale di Geofisica e Vulcanologia (INGV) and Laboratorio di Geofisica Sperimentale of the University of Firenze (LGS-UNIFI). Signals were also recorded by the Italian national seismic network at a range of hundreds of kilometres and by infrasonic arrays up to distances of 3700 km. Using state-of-the-art propagation modeling, we identify the various seismic and infrasound phases that are used for precise timing of the eruptions. We highlight the advantage of dense regional seismo-acoustic networks to enhance volcanic signal detection in poorly monitored regions, to provide timely warning of eruptions and reliable source amplitude estimate to Volcanic Ash Advisory Centres (VAAC).

## Introduction

Located in the Aeolian Islands, in Southern Italy, the Stromboli volcano (38.789N 15.213E, 920 m) is known worldwide for its persistent explosive activity of mild intensity from its open vent summit craters. Its ordinary explosive activity, repeating frequently at a rate of ~ 13 explosions/hour^1^, erupts scoria and ash up to a height of ~ 100–200 m above the craters, with ejecta fallout typically confined a circular crater of about 350 m diameter. This activity has been extensively studied with multiple geophysical observations, spanning from ground deformation and seismicity^2–5^, infrasound^6,7^, infrared thermometry^8,9^, doppler radar^10^ and videogrammetry^11,12^. The ordinary activity at Stromboli is occasionally punctuated by major explosions and paroxysms^13,14^. Paroxysms represent the largest-scale historical explosive events capable of generating convection plumes up to a height of 10 km and ejecting meter-sized blocks up to distances of 2 km from the vents reaching settled areas of Stromboli island. The catalog of paroxysms at Stromboli starts from 1879^13^. Since then, 23 paroxysms occurred before 2003 and 4 between 2003 and 2019^15,16^.

One of the main risks at Stromboli is due to tsunamis, which are able to affect not only the island but the whole coastal regions of southern Italy^14^. Caused by landslide or sector collapses, as well as pyroclastic flows entering the sea, at least 8 tsunamis related to Stromboli happened between 1879 and 2003^13,17^. On December 2002, a subaerial 11.6 Mm^3^ and submarine 9.5 Mm^3^ landslide at Stromboli^18,19^ triggered a tsunami wave that locally reached a height of 10 m, impacted the other Aeolian islands, and reached coastal regions of Southern Italy^17,20^.

Another risk associated with explosive eruptions is related to the emission of volcanic ash in the atmosphere. During an eruption, volcanic ash can reach and exceed the cruising altitudes of aeroplanes within minutes and spread over vast geographical areas within a few days. Encounters with volcanic ash may result in several problems such as malfunction or failure of engines. After some dramatic ash encounters by airplanes in the 1980’s, International Civil Aviation Organization (ICAO) has set up the International Airways Volcano Watch (IAVW). IAVW is based on 9 Volcanic Ash Advisory Centres (VAACs) designated by ICAO to provide near-real-time information on the largest possible number of volcanic events that affect aviation. Stromboli is located in the area of responsibility of the Toulouse VAAC, which includes a large part of Europe and Africa. In the IAVW chain of information, state volcano observatories play an essential role in providing to the VAACs near real time information on volcano activity from their monitoring networks.

Unexpected changes in explosion intensity at Stromboli are due to the dominant role of shallow conduit processes triggering paroxysmal activity^21^. On July 3rd and August 28th 2019, the volcano produced such paroxysms, followed by intense explosive and intermittent effusive activity. Visual observations and the analysis of the fall deposits allowed to characterize pyroclasts and reconstruct ballistic exit velocities of up to 160 m/s^22^. Paroxysms are driven by deep magma batches^23^ that eventually fragment in the shallow system producing the observed eruptive columns^24,25^. Short-term precursors of the July 3rd and August 28th 2019 paroxysms were identified^26^. The 2019 episodes consisted of large volcanic explosions, a few seconds apart, from different summit craters. The released eruptive column reached a height of ~ 5 km and produced a fall-out of lithic blocks, decimeter-sized scorias and ash affecting the summit areas as well as the inhabited settlements of the island. The collapse of the eruptive columns produced pyroclastic flows along the Sciara del Fuoco that entered the sea and triggered local tsunamis that reached wave heights of 1 m^27^.

Following the July 3rd explosions, the eruptive plume rose up the summit. The explosion was monitored in real time by two networks deployed and operated on the island by Laboratorio di Geofisica Sperimentale of the University of Florence (LGS-UNIFI)^28^ and Istituto Nazionale di Geofisica e Vulcanologia (INGV)^29^. With this event, INGV rapidly issued a volcano activity warning 30 min after the eruption through its 24/7 operating system (https://www.ct.ingv.it/index.php/monitoraggio-e-sorveglianza/prodotti-del-monitoraggio/comunicati-attivita-vulcanica). The Toulouse VAAC, then issued a Volcanic Ash Advisory (VAA) to the ICAO accounting for the presence of a volcanic ash cloud, drifting northerly. This VAA was based on satellite observations of the volcanic ash cloud.

On August 28th, short-time ground deformation precursors allowed an alert to be issued 5 min before the onset of the eruption^30^ and a Volcano Observatory Notice for Aviation (VONA) was sent by INGV about 30 min after the paroxysmal explosion, alerting for the presence of an ash plume rising up to 5 km above sea level (http://www.ct.ingv.it/index.php/monitoraggio-e-sorveglianza/prodotti-del-monitoraggio/comunicati-vona). A VAA was then issued by Toulouse VAAC announcing the presence of a drifting ash cloud in the vicinity of the volcano.

Infrasound observations can provide additional information about active volcanic processes^31^. The 2019 paroxysms were recorded at hundreds of kilometres across the permanent Italian seismological network operated by INGV, and in the far-field up to ~ 3700 km, by infrasound stations part of the International Monitoring System (IMS) completed by national arrays. At regional scales (e.g. beyond the first stratospheric returns at 150–200 km), combining observations from seismo-acoustic arrays allows improving operation monitoring methods to discriminate between natural and anthropogenic phenomena^32^. Johnson and Malone^33^ demonstrated that the source chronology and the timing of eruptions can successfully be calculated from ground-coupled airblasts observed at seismic stations. Analyzing records of dense seismic networks such as the transportable USArray^34^ or the European AlpArray^35^ provided unprecedented spatial detail of the infrasound ground footprint, allowing the detection and localization of both natural and man-made events with great precision. Using state-of-the-art modeling, the new era of massive datasets offers an opportunity to examine the propagation of infrasound wavefield across regional seismic network in more detail than previously possible and invert source information of a volcanic eruption.

In this study, we identify infrasound radiation from the 2019 eruptions recorded at both near- and far-field infrasound arrays as well as by seismic stations distributed across Italy. We show how far-field measurements are capable for providing timely warning to VAACs and estimating the source amplitude for remote volcanoes where local instrumentation is missing.

## Results

### Infrasound observations

The increased number of operating IMS stations and the establishment of regional infrasonic arrays demonstrate unprecedented potential of such an enhanced network in terms of detection capability, in particular for remote volcano monitoring^36,37^. In addition, national seismoacoustic monitoring systems have been developed in central Europe over several decades to fill a gap in the global IMS network^38^. Nowadays, the detection and location capabilities of combined networks offer a unique opportunity to investigate methods for discriminating between natural and artificial acoustic sources, as well as to better understand seismoacoustic coupling mechanisms at the Earth’s interface^39,40^.

**Figure 1:**
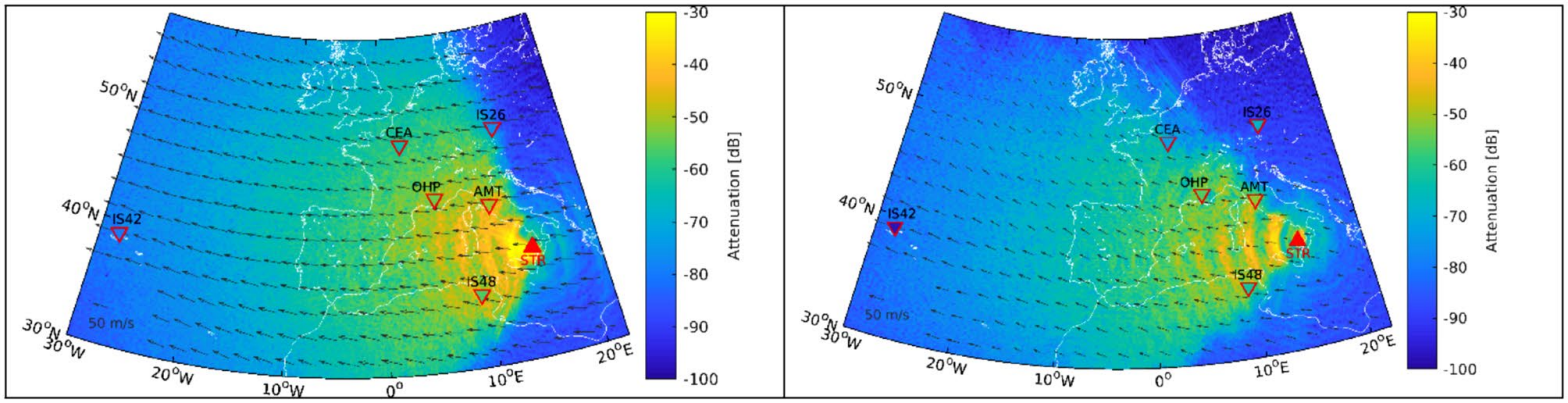
Attenuation maps derived from range dependent PE simulations at 1 Hz for the July 3rd (left) and August 28th (right) eruptions. Color-coded backgrounds represent the surface attenuation predicted by the PE model. The colored station markers represent attenuation of the array-observed amplitudes compared to a reference source amplitude at 1 km of 1510 Pa (July) and 466 Pa (August). For the sake of visibility, the northern most station IS37 is not included in the graphs. Table S2 provides details of the observed and modeled attenuation at all infrasound stations. The stratospheric wind field at 50 km altitude is represented by black arrows (50 m/s reference provided in the lower left corner). The map was generated using Matlab Mapping Toolbox Version 4.6 (https://www.mathworks.com/products).

Figure 1 shows the relevant infrasound network surrounding Stromboli over the Euro-Mediteranean region. At the time of the July 3rd and August 28th eruptions, easterly stratospheric wind flow prevailed at 30–60 km altitude and favored westward stratospheric propagation. Infrasound records at four IMS stations (IS26, IS42, IS37, IS48) and three national infrasound arrays (AMT, OHP, CEA) are analyzed. Table S1 highlights the detections for the July and August eruptions in short to medium distances (AMT: 543 km; IS48: 618 km; OHP: 977 km; IS26: 1124 km; CEA: 1509 km), as well as at long ranges (IS37: 3379 km; IS42: 3711 km), covering western (IS48, IS42) to northern (IS26, IS37) directions.

Figure S1 presents an example of infrasound detection at the IMS station IS48 for the July eruption. Wave parameters are calculated using the progressive multi-channel correlation method (PMCC) in the 0.1–4 Hz frequency band. Long lasting coherent waves are detected from 15:17 to 15:40 UTC. The increase with time of the apparent velocity (from 360 m/s to 380 m/s) indicates an increase of the ray turning heights at stratospheric altitudes. The frequency content of the detections is consistent with the low frequency (< 1 Hz) of infrasound produced by the paroxysm, that was followed by an intense spattering and explosive activity characterized by the higher frequency component (2–5 Hz) observed after 15:25 in the coda of the signals.

**Figure 2:**
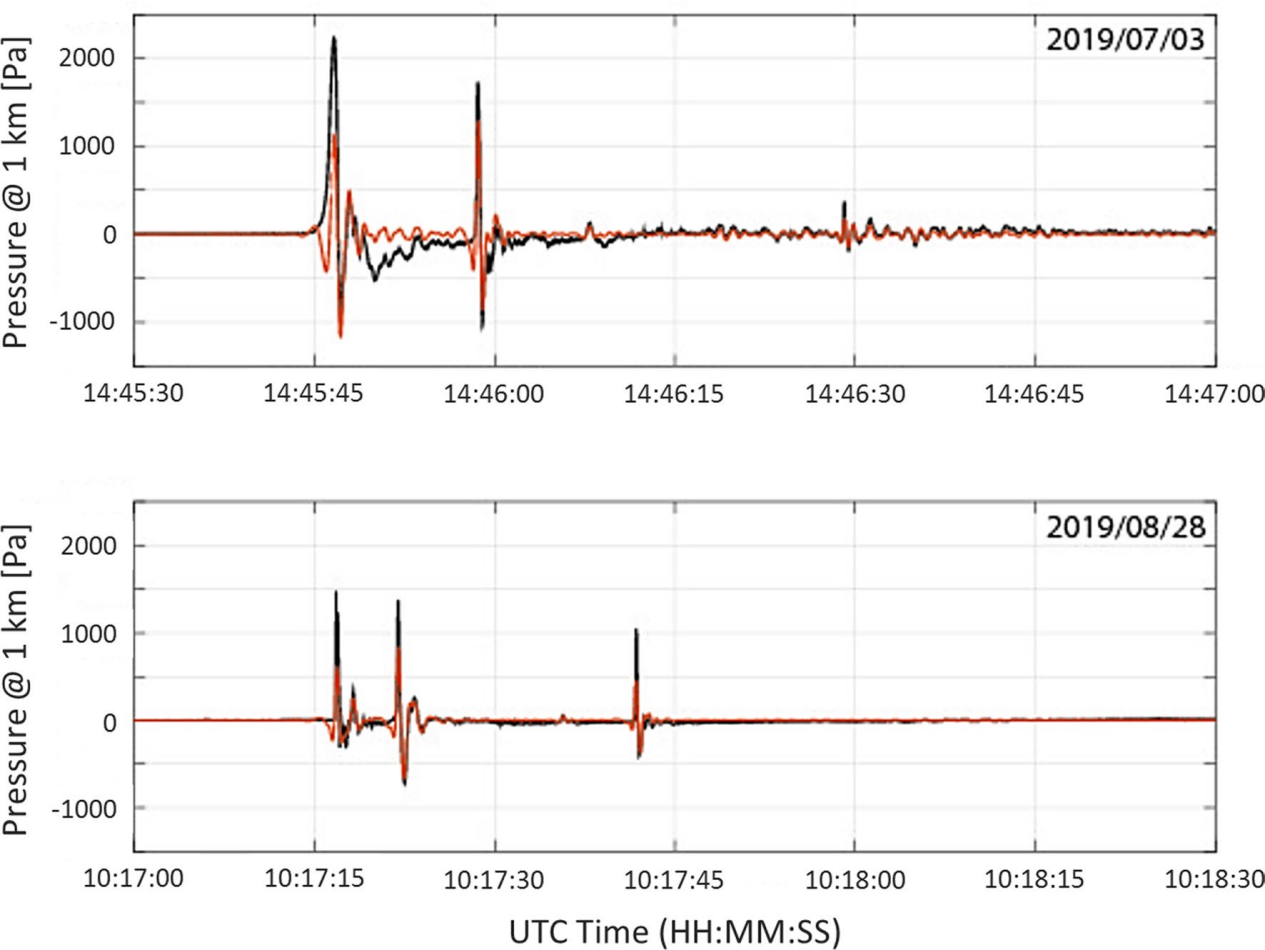
Raw infrasound pressure recorded at Stromboli for the July (top) and August (bottom) events reduced at 1 km from the source (black curve). Peak amplitudes of the first arrival reduce down to ~ 1300 and ~ 700 Pa respectively once band-pass filtered in the 0.5–2 Hz frequency band (red curve).

Table S1 summarizes the onset time, duration, back-azimuth, apparent velocity, frequency range and peak-to-peak amplitude of the detections. All arrivals are stratospheric, with average celerities ranging from 284 m/s to 312 m/s. Only the detection at IS37 in August might be thermospheric with a celerity of 267 m/s and arriving about 20 min later than would be expected for purely a stratospheric duct. Back-azimuth deviations from the true direction to Stromboli are all within 2°, except in the case of IS37 for the August eruption, which has a 6° deviation and is likely related to higher cross-winds affecting its southward propagation path. Figure 2 shows the raw infrasound pressure recorded at Stromboli by infrasound sensors operated by LGS-UNIFI for the July (a) and August (b) events at a reduced distance of 1 km from the source assuming a spherical geometrical spreading.

### Seismic observations

**Figure 3:**
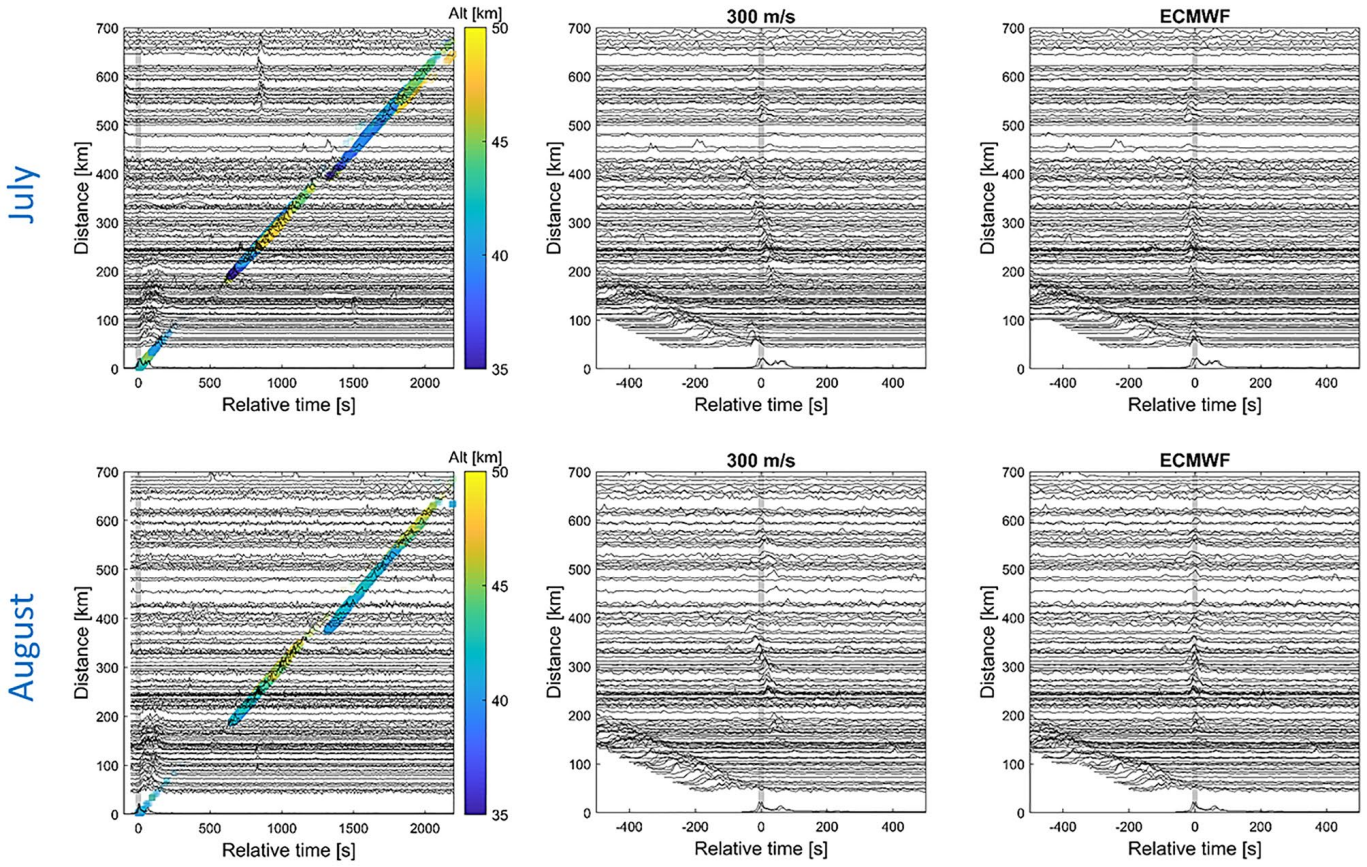
Hodochrons of seismic waveforms recorded at INGV seismic stations up to a distance of 700 km for the July (top, 198 stations) and August (bottom, 154 stations) eruptions. Time is relative to the main explosions (14:45:42 and 10:17:16 for the July and August eruptions, respectively). The envelopes of band-pass filtered data between 0.5 and 2 Hz are displayed. Left: distance versus relative time; theoretical arrival times derived from WASP-3D ray tracing simulations with turning heights color coded (in km). For the July eruption, the coherent seismic phases observed at a relative time of about 800 s for distances between 550 and 650 km show an apparent velocity consistent with a seismic phase. They are caused by a magnitude 1.8 earthquake that occurred at 16:59:30 UTC 33 km west of Siena, Tuscany (http://terremoti.ingv.it/event/22597531). Middle: time shifted traces using a constant celerity model of 300 m/s. Right: time shifted traces using celerity models derived from WASP-3D simulations.

Infrasound waves from the July 3rd and August 28th paroxysmal explosions were also detected by the permanent Italian seismological network up to a range of ~ 700 km. Figure 3 shows the time shifted seismograms according to a fixed celerity and modelled propagation times for stratospheric ducting (hodochrons in Fig. 1). Records exhibiting abnormal noise level likely related to sensor malfunction have been discarded. Time is relative to the origin of the main explosion (14:45:42 and 10:17:14 for the July and August eruptions, respectively) and are calculated from local infrasound records (Fig. 2) corrected for the source-to-receiver propagation time. Aligning the envelopes of band-pass filtered data between 0.5 and 2 Hz using a constant celerity of 300 m/s reveals the signature of stratospherically ducted air-to-ground phases up to a propagation range of ~ 600 km^34^.

In order to recover the source-time function of the eruption sequence from remote observations, a detailed analysis of the seismic records at stations located up to a distance of ~ 200 km from Stromboli is carried out. Figure S2 presents the seismic records, band-pass filtered between 2 and 8 Hz,. Out of the 38 available stations, impulsive signals are observed at four stations (MILZ, IFIL, MSRU, USI) with arrival times consistent with seismically coupled underwater acoustic waves (T-waves)^41^. These signals are associated with a seismic precursor which occurred at 14:44:44 UTC, about 1 min before the first explosion. The observed signals are not compressional P-waves usually generated by explosive events but hydroacoustic waves travelling at a velocity of approximately 1500 m/s, converted to seismic waves on the flanks of an island or on the continental coastal shelf. The travel times of the onset times of phases marked in Figure S2 is consistent with the expected velocity of T-waves.

### Long-range propagation modelling

For the July and August eruptions, 2D parabolic equation (PE) simulations clearly highlight several stratospheric bounces to the west with reduced attenuation (Fig. 1) and are in agreement with the summer conditions of the predominant westward stratospheric wind. The first five to ten ground returns of stratospheric waves are visible as concentric rings of reduced amplitude arrivals to the west of Stromboli reaching arrays AMT, IS48 and OHP. These stations are all within 1000 km of Stromboli and are in a range of azimuths where stratospheric returns are modeled with attenuation values between 50 and 65 dB. At greater distances, stations CEA, IS26 and IS42 capture stratospheric phases with higher attenuation values between 65 and 95 dB, while IS37 located at 3379 km to the edge of stratospheric ducting arrivals has attenuation between 100 and 110 dB.

Reference source amplitudes ranging from 1 Pa to 10 kPa with 1 Pa steps are applied to define the respective observed attenuations at the different stations. This reference is estimated by applying a least square fit between the modeled and observed attenuation values at seven stations (Table S2). The minimized quadratic difference occurs for a source amplitude of 1510 Pa on July 3rd and 466 Pa on August 28th with standard errors of 8 dB and 14 dB respectively when considering all observations.

The converted station attenuations deduced from the latter results are in the range of 50 to 65 dB for AMT, IS48 and OHP, between 65 and 85 dB for CEA, IS26 and IS42 (excluding the low amplitude station observation of IS42 in August) and 90 to 115 dB for IS37. This method of deriving source signal intensity independently from near-source observations yields results consistent with near-field measurements peaking at ~ 1300 and ~ 700 Pa respectively once band-pass filtered in the 0.5–2 Hz frequency band of analysis of long range infrasound data (Fig. 2).

Figure 3 presents the hodochrons of the seismic waveforms recorded at INGV seismic stations up to a distance of 700 km from Stromboli for the July and August eruptions. Superimposed on the left panels are theoretical arrival times of direct (up to ~ 80 km, celerity of 340 m/s) and stratospheric phases (first branch ranging from ~ 200 km to ~ 400 km and second branch ranging from ~ 400 km to ~ 650 km; celerities between 270 m/s and 305 m/s). At almost all stations, the maximum of the signal amplitude coincides with the first and second stratospheric branches with ray turning heights ranging between 35 and 50 km. The summer atmospheric conditions of July 3rd and August 28th are comparable. The mean time residuals (mean of the difference between the observed and modeled propagation times) reduce from 20 ± 4 s when using a constant celerity of 300 m/s to 5 ± 2 s when accounting for a variable model-based celerity.

**Figure 4:**
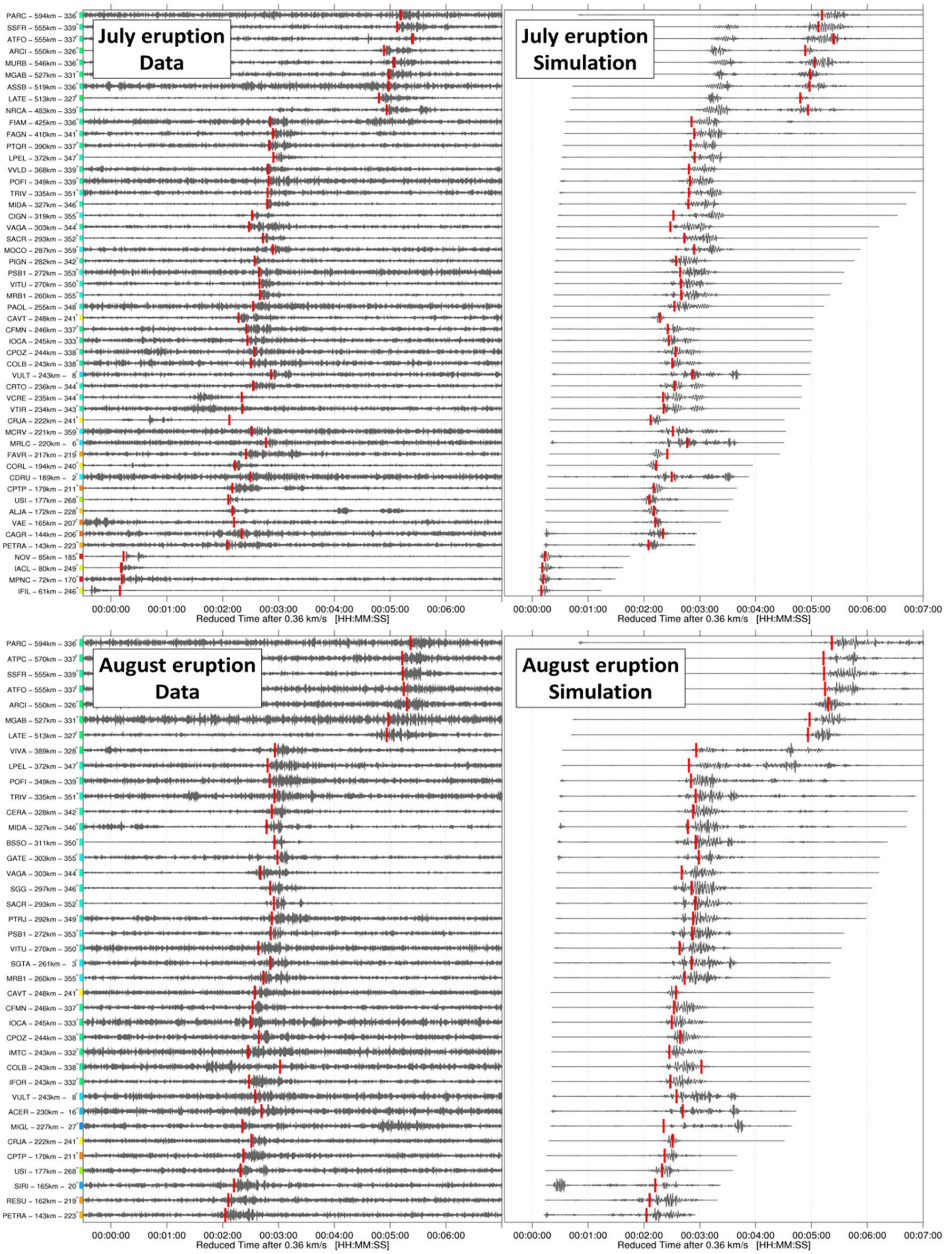
Comparison between the recorded (left panels) and the modeled (right panels) waveforms at seismic stations for July (top panels) and August (bottom panels) eruptions. Only stations with clear signals (SNR > 10 dB) are displayed (50 and 38 stations for the July and August eruptions, respectively). Station names, distances and azimuths are specified to the left. Waveforms are time-shifted using a constant celerity of 300 m/s. Vertical red bars are onset times of dominant arrival at each station used for the localization (Figs. 4 and 5), performed on the recorded waveforms band-pass filtered between 0.5 and 2 Hz. To ease the comparison between data and simulation, picks are reported on the modeled waveforms (right). A good agreement beween the predicted and observed arrival times, but also shapes and durations is observed.

Figure 4 compares the recorded and modeled infrasound waveforms obtained from normal mode simulations^42^ of the wave equation for the July and August eruptions. For each station, range-dependent effective sound speed profiles are considered. The source time function used to model synthetic waveforms is the zero-phase Ricker wavelet with a dominant frequency of 1 Hz. Overall, a good agreement between the observed and predicted arrival times and signal duration is observed. Considering the onset times of dominant arrivals (one per station), the mean arrival time residual is 4.8 ± 0.5 s. Beyond 500 km, dominant arrivals switch from the first to the second stratospheric branch for both events. In the observations, the first stratospheric branch does not extend beyond ~ 500 km, as predicted for the August eruption. For the July eruption, the propagation simulation extends the first stratospheric branch up to ~ 600 km; this effect can result from inaccurate atmospheric specifications^43^ or unrealistic gravity wave fields diffracting acoustic energy into the geometric shadow zone^44^.

### Location accuracy

**Figure 5:**
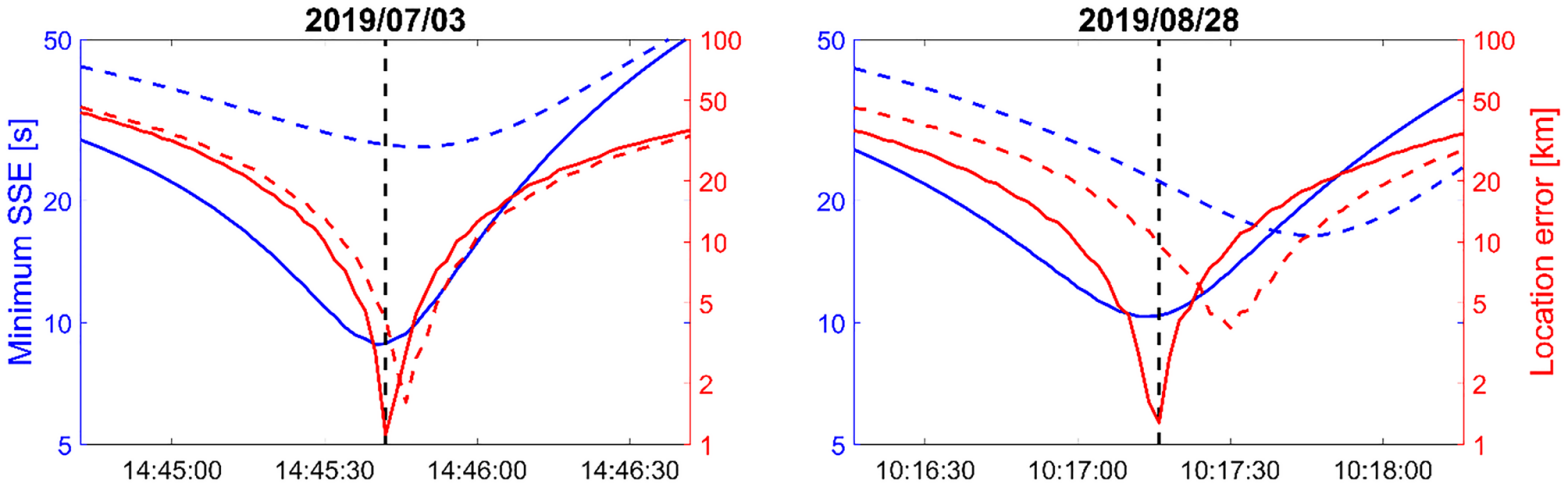
Sum of squared errors (SSE) (blue curves) and location errors (red curves) for the July (left) and August (right) eruptions as a function of trial origin times. Dashed and solid lines correspond to a constant celerity (300 m/s) and azimuthal/range dependent celerity models derived from WASP-3D ray tracing simulations, respectively.

Figure 5 shows the time residuals (SSE) for both July and August eruptions considering a constant celerity of 300 m/s and travel times derived from 3D ray tracing simulations. No assumption is made on the event location. The search region extends from 36 to 44 N and 10E to 20E with a grid resolution of 0.5 km. The travel times are calculated over a time period of 2 min centered on the origin time of the paroxysmal eruptions with a time step of 1 s (Figure S2). The minimum SSE is calculated over the studied region (blue curves), from which the distance misfit between the candidate source point and Stromboli is calculated (red curves). Using a constant celerity of 300 m/s, the minimum of SSE is reached at 14:45:48 UTC on July 3rd and 10:17:46 UTC on August 28th, with corresponding errors of 6 s and 32 s compared with the true origin time. When considering modeled celerity values using atmospheric models from the European Centre for Medium-Range Weather Forecasts (ECMWF IFS cycle 38r2, http://www.ecmwf.int), the origin time error reduces to ~ 2 s (14:45:40) in July and ~ 3 s (10:17:13) in August, and the corresponding location error decreases from 1.6 km to 1.1 km in July, and 3.7 km to 1.3 km in August.

**Figure 6:**
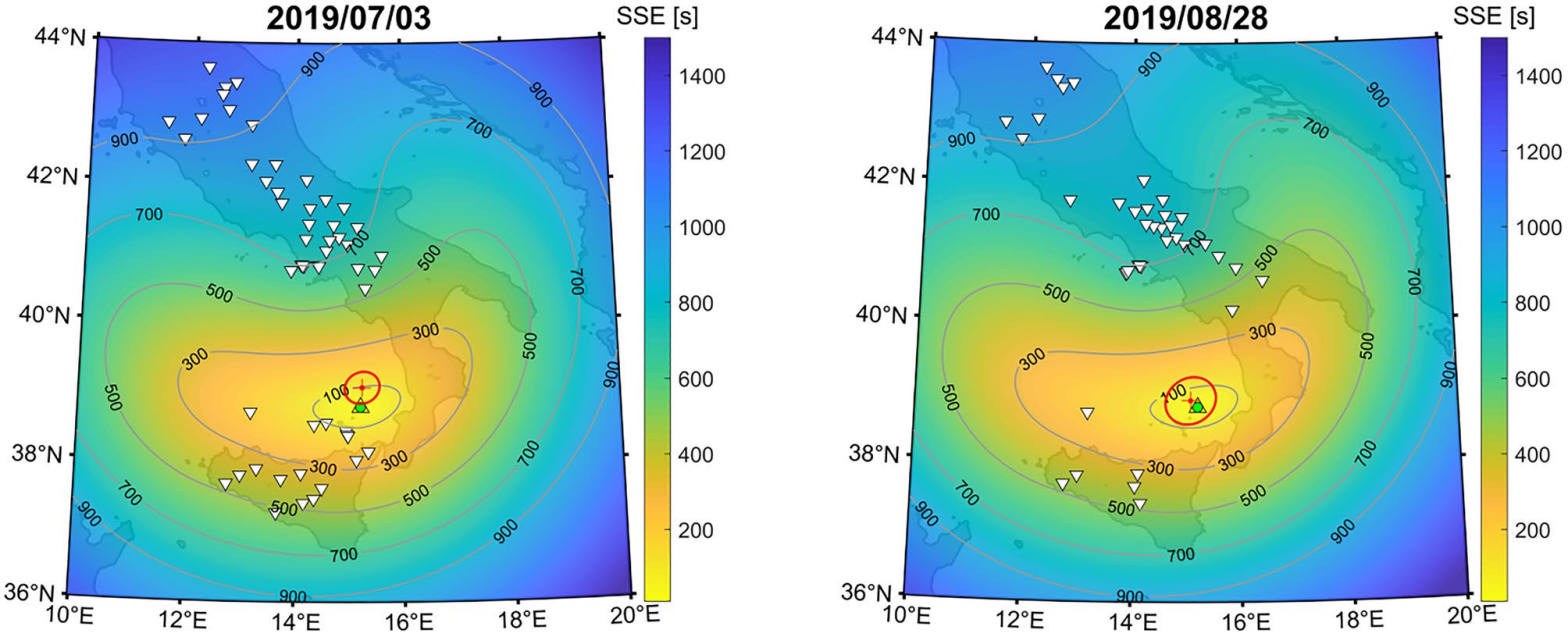
Geographical distribution of the time residuals for the July (left) and August (right) eruptions using the origin time given by the minimum of SSE. Grey curves are isocontours of SSE. White inverted triangles are selected seismic stations where clear signals from the eruptions (SNR > 10 dB) were observed (50 and 38 stations for the July and August eruptions, respectively). The red crosses indicate the centroid of cross-bearing location considering wind-corrected back-azimuths at stations IS26, IS37, IS42, IS48, AMT, OHP and CEA. The red ellipses show the 95% confidence. The map was generated using Matlab Mapping Toolbox Version 4.6 (https://www.mathworks.com/products).

Figure 6 presents the geographical distribution of the time residuals for the July and August eruptions. Time residuals are calculated using the origin time given by the minimum of SSE (Fig. 5, solid blue curve). Due to the geographical distribution of the stations, the residual contours are elongated east–west and location uncertainty is greater in this orientation. For comparison, the locations obtained using back-azimuths from seven infrasound arrays only are indicated by the red crosses. The red ellipses show the 95% confidence location using wind (ECMWF IFS) corrected back-azimuths (Table S1) and a measurement uncertainty of 1° appropriate for arrays of kilometre-scale^45^. When considering locations derived from infrasound arrays only, the source is located 31 km and 16 km north of Stromboli for the July and August eruptions, respectively.

### Notification of volcanic activity

During the last decade, the deployment of infrasound stations at local, regional and global scales has significantly increased the potential of infrasound measurements to mitigate volcanic hazards^46^. In particular, it was demonstrated that infrasound can play a key role in supporting warning systems by notifying, in real-time, the onset of explosive eruptions^47^, thus reducing the risk of aircrafts encountering volcanic ash in poorly monitored regions. The amplitude of infrasound detections associated with the July and August eruptions is corrected for propagation effect along each source-to-receiver pathto infer the source amplitude at a reference distance of 1 km from the crater^48^. The source amplitude is then multiplied by the normalized number of detections per minute, which reflects the time persistency of the signals recorded at the array.

Applying the procedure described by Ulivieri et al.^49^ and Ripepe et al.^47^, the occurrence of significant activity at Stromboli is estimated from remote infrasound observations. Infrasound detections at all arrays are filtered according to the true back-azimuth to Stromboli (± 10°), band-pass filtered from 0.5 to 2 Hz, and the mean amplitude (P) is calculated in a sliding window of 20 min. The time persistency of the signals is quantified by the number of detection per minutes (Ndt), normalized to 60. The mean amplitude (P) is corrected for propagation effect along each source-to-receiver paths in order to infer the source amplitude at a reference distance of 1 km from the crater (Ps). The source pressure and the signal persistency are then combined to derive the infrasound parameter (IP = Ps*Ndt) that is used to identify significant ongoing volcanic activity^47,49^. A notification is obtained when IP exceeds a threshold set to 60 over a period of at least 20 min^50^.

**Figure 7:**
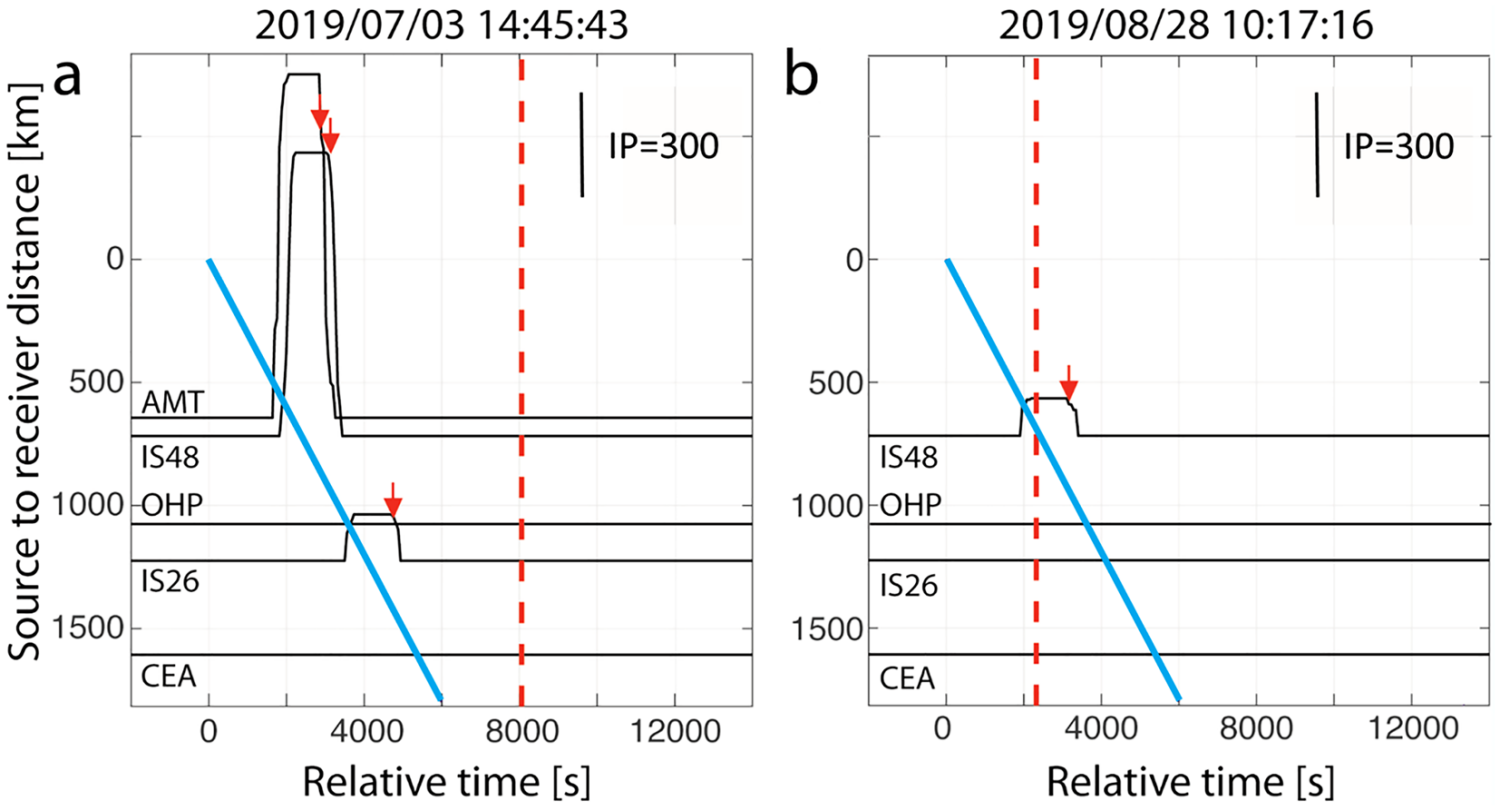
IP derived from infrasound detections at several stations for the July 3rd (**a**) and August 28th, 2019 (**b**) paroxysms at Stromboli. The vertical red line identifies the time of issuance of the VAA from Toulouse VAAC, while the pink line indicates the 300 m/s travel-time typical of stratospheric arrivals. The onset time of increased volcanic activity, based on the IP values exceeding the threshold of 60 for at least 20 min at each array, is shown by the red arrows. Such notification would have been possible at AMT, IS48 and IS26 for the July event, and at IS48 for the August event.

Figure 7 shows the IP variations at stations AMT, IS48, OHP, and IS26. IP values exceeding the threshold of 60 are highligted.. By applying such an approach, notifications could have been delivered up to a distance of 1124 km (IS26) and 618 km (IS48) for the July and August events, respectively. For information purpose, the July 3rd notification based on infrasound observations at the closest IMS array in Tunisia would have preceded in the best case the VAA alert issued at 17:00 UTC by 105 min. On August 28th, a VONA was sent by INGV at 10:48 UTC, 31 min after the eruption, allowing a VAA to be issued by Toulouse VAAC at 10:56 UTC. In this case, the first infrasound based notification using records at IS48, would have been possible at 11:10 UTC. It should be noted that no location can be computed using a notification based on a detection at single array, as opposed to more detailed source information derived from the infrasound wavefield analysis across a dense network. The level of confidence of such notification should therefore account for the station detection capability and its sensitivity to local environmental noise. The added value of regional seismic network to contribute to an alert system in poorly monitored regions would require further evaluation and benchmark testing which are out of the scope of this paper.

## Discussion

The 2019 paroxysmal eruptions of Stromboli were clearly detected by IMS infrasound stations up to a distance of 3711 km. In addition to the IMS network, signals were also recorded by the national seismological network across Italy. The infrasound signature of the eruptions as measured by infrasound and seismic sensors has been modeled at local and regional distances (direct/tropospheric versus stratospheric returns). The amount and variety of observed infrasound phases represents a unique dataset for statistically evaluating atmospheric models, numerical propagation modeling and localization methods.

At distance of about 170 km from Stromboli, transient signals were observed at four seismic stations (MILZ, IFIL, MSRU, USI) with arrival times consistent with T-waves. The timing of the T-waves is not consistent with the paroxysmal explosion but rather to a shallow volcano-tectonic earthquake reported by Giudicepietro et al.^26^ which occurred at 14:44 UTC, one minute before the July 3rd eruption. The lack of T-wave observations at other stations is explained by a masking effect (see map on Figure S3).

A modeled estimate of the ground return footprint of stratospheric arrivals is recovered using 2D PE and ray-tracing simulations, as well as normal mode simulations. The model-predicted and array-observed infrasound attenuation ranges correspond to values within ± 10 dB for July and August (Table S2). The average deviation in dB between model and observation are 3.4 dB for the July eruption and 8.7 dB for the August eruption. The higher accuracy of the modeling on July 3rd is explained by a more stable and favorable ducting condition, lowering uncertainty in modeling the amplitude^51^. In August, the uncertainty between model prediction and observation is especially high at IS26, as the station is located outside of the modeled stratospheric cone. By minimizing the quadratic difference between the observed and predicted attenuation, a source amplitude of 1510 Pa on July 3rd and 466 Pa on August 28th is inferred. This independently derived source amplitude is consistent with near field observations when modeling long-range propagation at 1 Hz and band-pass filtering records around the dominant frequency of the signal (0.5–2 Hz) (Fig. 2).

Using only IMS infrasound detections, the cross-bearing location shows errors up to ~ 30 km. Using the arrival times of air-to-ground coupled waves and celerity models derived from 3D ray-tracing simulations reduce the origin time error down to ~ 3 s. These results underline the benefit of integrating local and regional infrasound networks as well as seismic networks into the global IMS network for improving location and discrimination methods which are used as effective tools for the CTBT verification regime.

This study also highlights the added value of exploiting the synergy between complementary networks to develop efficient multi-technology monitoring systems for disaster prevention or mitigation^52^. It should be emphasized that volcanic signals recorded by remote seismo-acoustic monitoring systems are less valuable due to reduced signal-to-noise ratio and propagation time (e.g. latency of ~ 15 min at 300 km). Furthermore, while infrasound is recorded on seismic stations up to distances of 700 km, the seismic signal produced by the explosion is limited to stations located within ~ 250 km, thus limiting the use of seismic waves only to local and regional distances. However, as infrasound propagate downwind with reduced attenuation, recent advances in network performance modeling allows optimizing network layout in order to best detect and characterize volcanoes^53^. The reverse time migration method, which was successfully implemented by Walker et al.^54^, would be highly beneficial to automatically detect infrasonic signals registered by a dense seismo-acoustic network and locate the source. Compared with detection at single infrasound array, such an approach increases the confidence level of the notification by discarding false events along the direction of the targeted volcano. Following the detection stage, propagation modeling using a realistic description of the atmosphere would provide a precise source location reducing false alarms associated with detection timing of the eruptions and reliable source amplitude estimate. The real-time reliability of the reverse time migration method would require further statistical performance testing to define acceptable detection thresholds (e.g. SNR, number of detections) to be used under realistic operating conditions.

Within recent years, the development of cost-effective acoustic sensors offers an opportunity to examine the propagation of infrasound wavefields, in high resolution, across a dense scalable network^55^. Traditional methods used to monitor volcanoes have inherent drawbacks for volcanic hazard assessment (e.g. local monitoring sensors are vulnerable during large volcanic eruptions and ash plumes below cloud cover are difficult to detect using satellite remote sensing). The infrasound technology offers an alternative tool to mitigate volcanic hazards by providing not only the timing of the eruption chronology by also estimate of the plume height which is a key input parameter for running ash dispersion models. Such technological advances, associated with improved processing and propagation modeling methods, share the advantages of being non-invasive and capable of exploring wide areas of investigation. This geophysical exploration technique is particularly beneficial to monitor volcanic regions with little infrastructure, being not limited by field accessibility. The effectiveness of such method has been demonstrated at propagation ranges of thousands of kilometres through eruption case studies in Southeast Asia where there are around 750 active or potentially active volcanoes^56^. It is expected that pursuing such evaluation would contribute to improving methods for natural hazards monitoring from the perspective of building a timely warning system to VAACs in poorly monitored regions.

## Methods

### Data processing

The PMCC was applied to the raw pressure sampled at 20 Hz to detect coherent infrasound signals within the background noise and estimate the wave front parameters^57^. The time and frequency window parameters for PMCC processing were set using 1/3-octave band scheme in the 0.1–5.0 Hz band to facilitate detection of broadband signals. Window time durations were set to vary linearly with the period, with a 90% overlap. Such log-scale configuration with variable window length improves discrimination between interfering signals^58^. At a sampling rate of 20 Hz, the resolution of the azimuth is about 1° and 5 m/s for the horizontal trace velocity^43^.

To quantitatively determine the optimum source location using the arrival time of air-to-ground coupled waves observed across the Italian seismic network, a grid-search method for a single-source model is implemented. The optimum location is estimated by calculating the normalized sum squared errors (SSE) of the misfit between the observed and predicted arrival times at seismic stations^59^.

### Propagation modelling

To predict the pressure wave attenuation, infrasound propagation is modeled using a two-dimensional parabolic equation approach (2D PE) up to distances of 4500 km from Stromboli^60^. Simulations are computed covering the full azimuth range with a step width of 0.1°. Atmospheric variability is taken into account using time-varying atmospheric models. The used ECMWF IFS analysis products contain gravity waves (GWs) originating from different sources such as orography, convection, wind shears, jets and fronts. IFS captures well the synoptic scale meteorology below ~ 30 km altitude. However, activity and interaction of GWs with the mean flow are not predicted accurately due to imperfect parameterisations^61^, or the lack of fine scale orography leading to underestimated or missing GW activity^62^, for instance. The lack of assimilated observations at stratospheric altitude and a sponge layer starting already in the stratosphere are also responsible for significant temperature and wind biases in the middle atmosphere^43^. In order to account for unresolved wind perturbations, small scale atmospheric variations from the spectral gravity wave model developed by Gardner et al.^63^ are added to the ECMWF atmospheric fields. Such empirical corrections significantly affect infrasound propagation in the stratopause region where ray-turning heights are quite sensitive to small-scale atmospheric perturbations^51^. This approach was already successfully employed to explain explosion detections through diffraction and scattering of acoustic energy into the geometric shadow zone across Europe^44^.

For identitying the propagation paths of the recorded signals, long-range infrasound propagation is simulated using the windy atmospheric sonic propagation ray theory-based method (WASP-3D) which accounts for the longitudinal variation of the atmospheric model along the propagation paths^64^. The ray canonical variables (slowness vector, position, and propagation time) are numerically solved by linearized hydrodynamic equations in spherical coordinates. Following a shooting procedure, the azimuthal deviations of eigenrays sought between Stromboli and infrasound arrays are calculated. Rays are classified and labeled depending on their turning heights and number of ground reflections before reaching the station. Extracted celerity models and azimuthal deviations are median values among the selected rays. This procedure, which is preferable to costly eigenray techniques, yields reliable station-dependent travel times and azimuthal corrections needed for accurate source localization^40^.

Synthetic waveforms are also calculated at seismic stations where the signal-to-noise of the observed air-to-ground coupled signals allows unambiguous arrival time phase picking. The full-wave propagation method used is a low order range-dependent propagation model obtained from a normal mode wavelet-based decomposition of atmospheric perturbations^42^. Selecting the components leading to the most sensitive eigenvalues yields accurate simulations with significantly reduced computational cost.

## Supplementary Information

Supplementary Information.

## Data Availability

Seismic records were obtained from the national seismic network operated by INGV (http://esm.mi.ingv.it, last accessed August 2020). The vertical component data of broadband seismic stations were downloaded from the INGV International Federation of Digital Seismograph Networks (FDSN) web services (http://terremoti.ingv.it)^65^. ECMWF products, including the atmospheric model analysis are available via https://www.ecmwf.int/en/forecasts/dataset (last accessed August 2020) under CC-BY 4.0 License. Bathymetry and topographic data are from the Shuttle Radar Topography Mission (SRTM30) digital elevation model (DEM) (http://eros.usgs.gov). The DTK-GPMCC software included in the NDC-in-a-Box package designed for infrasound array processing is available upon request. Infrasound records at IMS and national arrays can be found at the BGR data product center (https://produktcenter.bgr.de/terraCatalog/DetailResult.do?fileIdentifier=ef5bd209-a3a1-44de-b8c6-ec5e7a0dce76).
