# Supplementary material for: Using dense seismo-acoustic network to provide timely warning of the 2019 paroxysmal Stromboli eruptions
Source: Sci Rep. 2021 Jul 14;11:14464. doi: 10.1038/s41598-021-93942-x (PMC8280234; doi:10.1038/s41598-021-93942-x)
Supplement: Supplementary file 1 — Supplementary Information. [file 41598_2021_93942_MOESM1_ESM.docx]

**Using dense seismo-acoustic network to provide timely warning of the 2019 paroxysmal Stromboli eruptions**

^1*^A. Le Pichon, ^2^C. Pilger, ^2^L. Ceranna, ^3^E. Marchetti, ^3^G. Lacanna, ^1^V. Souty, ^1^J. Vergoz, ^1^C. Listowski, ^1^B. Hernandez, ^1^G. Mazet-Roux, ^1^A. Dupont, ^4^P. Hereil

*^1^CEA/DAM/DIF, F-91297, Arpajon, France*

*^2^BGR, B4.3, D-30655 Hannover, Germany*

*^3^University of Firenze, Department of Earth Sciences, 50121, Firenze, Italy*

*^4^Meteo France, Toulouse VAAC, 31057 Toulouse, France*

*^*^Corresponding author: alexis.le-pichon@cea.fr*

# Supplementary Information

# This section contains a description of the infrasound signal parameters (Table S1) and a comparison between the observed and modeled attenuation values at infrasound arrays (Table S2). Figure S1 shows an example of PMCC detection at IS48. Record section of INGV seismic stations is shown on Figure S2. Figure S3 shows a map of seismic stations in a range of 200 km from Stromboli volcano.

**Table S1:** PMCC results for IMS and national infrasound arrays. Values are indicated in black and red for the eruptions of July 3^rd^ and August 28^th^, respectively. Peak-peak amplitudes are measured on the best beam of signals band-pass filtered between 0.5 and 2 Hz.

| Station | Distance  [km] | True back-azimuth  [deg] | Onset Time  [UTC] | Duration [s] | Wind-corrected back-azimuth  [deg] | Apparent velocity  [m/s] | Frequency  range  [Hz] | Peak-peak amplitude  [Pa] |
| --- | --- | --- | --- | --- | --- | --- | --- | --- |
| AMT (2 channels) | 543 | 145 | 15:13:24  - | 370  - | -  - | -  - | 0.4-2  - | 2.8  - |
| IS48 | 618 | 56 | 15:16:50  10:51:00 | 400  345 | 55  53 | 365  355 | 0.2-5  0.2-5 | 1.84  0.29 |
| OHP | 977 | 122 | 15:39:30  11:08:50 | 120  780 | 122  121 | 360  370 | 0.3-2  0.4-3 | 0.42  0.61 |
| IS26 | 1124 | 173 | 15:44:50  11:17:00 | 440  830 | 173  175 | 355  350 | 0.1-4  0.1-5 | 0.17  0.14 |
| CEA | 1509 | 131 | 16:01:00  11:38:00 | 800  360 | 132  129 | 355  355 | 0.4-3  0.5-2 | 0.32  0.45 |
| IS37 | 3379 | 185 | 17:54:20  13:47:30 | 110  55 | 190  180 | 335  350 | 0.6-3  0.6-2 | 0.01  0.01 |
| IS42 | 3711 | 76 | 18:09:40  13:34:40 | 410  65 | 76  73 | 350  350 | 0.2-3  1-3 | 0.12  0.01 |

**Table S2:** Comparison of observed (dB values derived from least square approximation of source intensity) and modeled (using 2DPE propagation model) attenuation values in dB.

| ***Station*** | ***July, 3^rd^ observed*** | ***July, 3^rd^ modeled*** | ***August 28^th^ observed*** | ***August, 28^th^ modeled*** |
| --- | --- | --- | --- | --- |
| *AMT* | *-53.6* | *-53.2* | *-54.6* | *-64.2* |
| *IS48* | *-58.3* | *-52.5* | *-64.1* | *-57.4* |
| *OHP* | *-56.5* | *-57.5* | *-58.1* | *-58.1* |
| *IS26* | *-78.9* | *-85.7* | *-70.6* | *-91.4* |
| *CEA* | *-66.9* | *-66.9* | *-75.3* | *-71.3* |
| *IS37* | *-110.9* | *-105.9* | *-91.4* | *-102.3* |
| *IS42* | *-82.1* | *-87.1* | *-100.3* | *-91.7* |


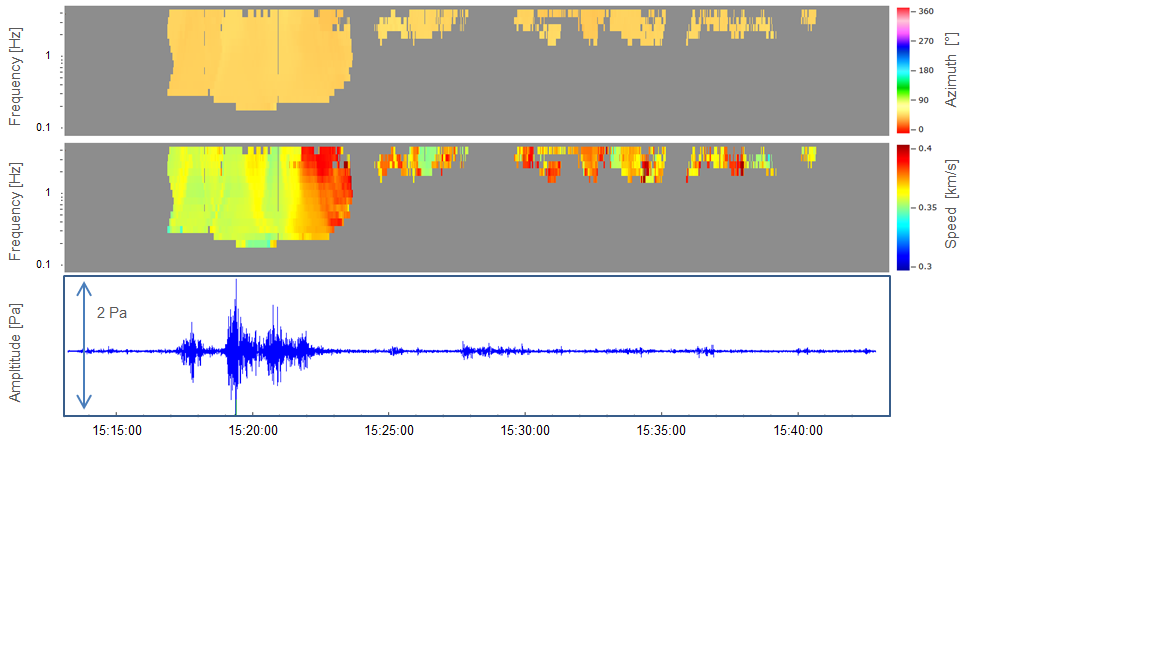
Figure S1 – Results of PMCC processing at the IMS station IS48. Top: back-azimuth (clockwise from North). Middle: apparent velocity. Bottom: infrasound recording at the central array element band-pass filtered in the 0.5-2 Hz frequency band.


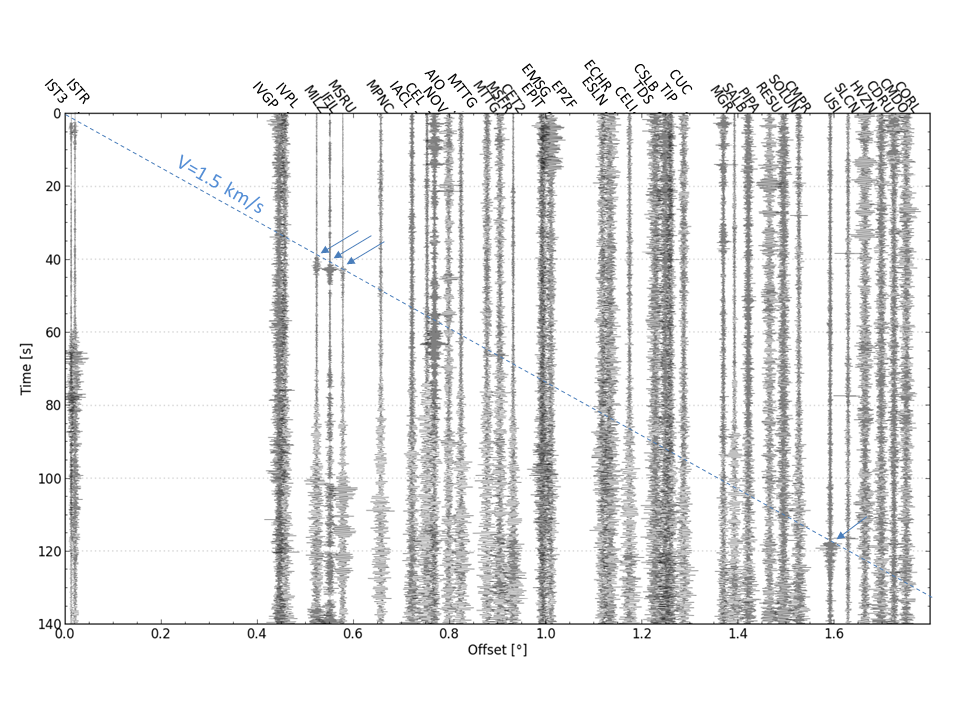


Figure S2 – Record section of INGV broadband vertical seismic stations. Data are band-pass filtered between 2 and 8 Hz with an epicentre located on the summit of Stromboli volcano at an origin time of 14:45:42 UTC on July 3^rd^ 2019. Blue dashed line represents an apparent velocity of 1.5 km/s corresponding to the celerity of hydroacoustic waves. Blue arrows point to the T-waves observed at MILZ, IFIL, MSRU and USI.

*
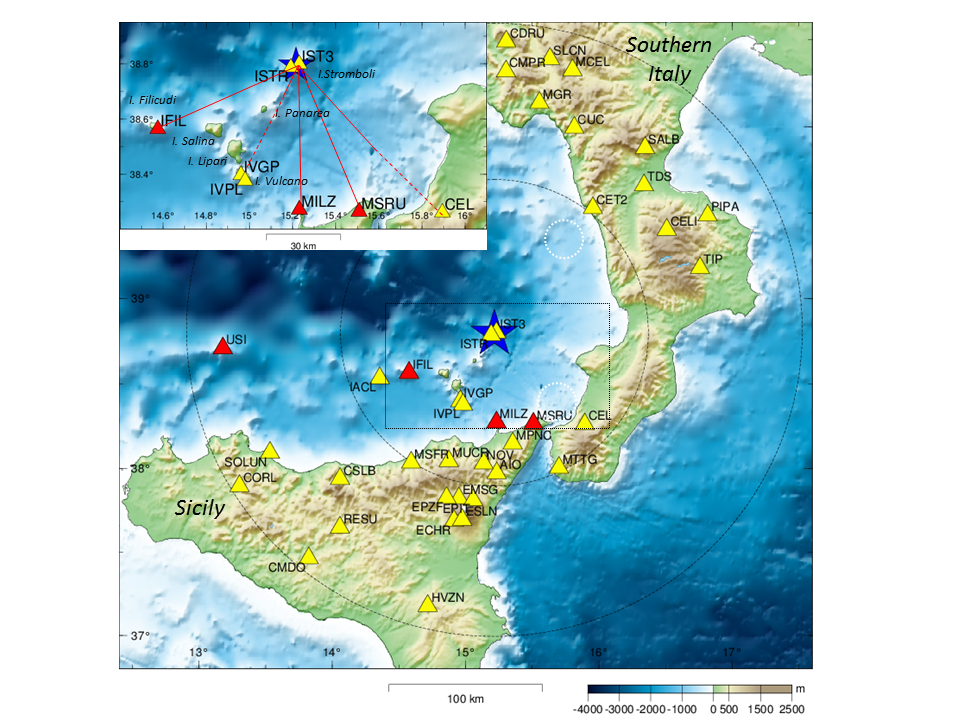
*

Figure S3 – Map of INGV broadband seismic stations. Black dashed circle show radius of 100 km and 200 km from Stromboli volcano, respectively. Red triangles are stations that recorded impulsive signals consistent with T-wave arrivals. White dashed circles indicate shoals that could prevent T-waves reaching seismic stations along the coastline. Blue star indicates the Stromboli volcano. Panarea and Filicudi Islands may have blocked or diffracted energy such as T-waves could not reach stations IVPL, IVG and IACL (see inset on Figure S3) while the presence of shoals between Stromboli and the southern Italian coastlines may have reduced the coupling efficiently into P-waves. The map was generated using GMT 5.4.3 (https://www.generic-mapping-tools.org) with GEBCO 2020 global terrain model for ocean and land at 15 arc-second intervals (https://www.gebco.net/data_and_products/gridded_bathymetry_data).
